# Reproductive Toxicity of *Theobroma cacao*: Increase in Survival Index, Nongenotoxic, and Proimplantation Potential

**DOI:** 10.1155/2021/6114672

**Published:** 2021-01-12

**Authors:** I. J. Asiedu-Gyekye, T. G. Borovskaya, M. E. Poluektova, А. V. Vychuzhanina, Y. А. Shchemerovа, S. I. Kamalova, V. A. Grgoreva, P. Amoateng, K. E. Kukuia, A. A. Kwapong, L. Allotey Babington, S. K. Amponsah, B. B. N'guessan

**Affiliations:** ^1^Department of Pharmacology and Toxicology, School of Pharmacy, College of Health Sciences, University of Ghana, Legon, Accra, Ghana; ^2^Goldberg Research Institute of Pharmacology and Regenerative Medicine, Tomsk, Russia; ^3^National Research Medical Center of the Russian Academy of Sciences, Tomsk, Russia; ^4^Department of Pharmaceutics and Microbiology, School of Pharmacy, College of Health Sciences, University of Ghana, Legon, Accra, Ghana

## Abstract

Unsweetened natural cocoa (UNCP) was evaluated for reproductive toxicity in rats. A preliminary genotoxic potential was evaluated by the DNA comet assay test using C57Bl/6 mice. Both therapeutic dose (TD; 900 mg/kg) and high dose (HD; 9000 mg/kg) of UNCP were used. White Wistar rats were used in two experimental groups. The females received UNCP 15 days before crossing with untreated males. The males received UNCP for 48 days before mating with untreated females. Subacute toxicity was observed during a 14-day oral administration of UNCP. Results show that a high tail DNA% was observed with methyl mesylate administration in all tissues analysed. The lowest tail DNA% value was observed in the liver (1.64 ± 0.26) and kidney (1.63 ± 0.30) during UNCP (TD) administration. UNCP did not induce observable physical congenital malformations on the pubs of treated female and male rats, lacks genotoxic potential, and did not adversely affect pregnancy index, pub weights, and survival index, but UNCP exhibited proimplantation potential (*p* > 0.05).

## 1. Introduction

In Ghana, herbs are used in medicine and food in various forms, especially those used in enhancing sexual and reproductive functions. One of such herbs is *Theobroma cacao* in the form of UNCP [1–3].

Unsweetened natural cocoa powder (UNCP) is a pulverised high-grade nonalkalized powder used as beverage in Ghana, West Africa, and most parts of the world. UNCP is obtained from the seed of *Theobroma cacao* and is prepared after removal of the cocoa butter from powdered cocoa beans via fermentation, drying, bagging, winnowing, roasting, grinding, and pressing. After extraction and compression, the solid blocks obtained are pulverised into a very fine powder. The pharmacological and health benefits of UNCP have been previously studied [1–3]. UNCP has been recommended in the management of simple uncomplicated malaria, bronchial asthma, essential hypertension, diabetes mellitus, and as an aphrodisiac [4–7].

The cardioprotective, hepatoprotective, antiasthmatic, and aphrodisiac potentials of UNCP (*Theobroma cocoa*) place this nutraceutical in a likely situation and lead to its excessive consumption among consumers. Moreover, toxicity studies on this nutraceutical are rare. There are no documented and visible adverse effects of UNCP. The common notion is that medications and nutraceuticals of plant origin are comparatively safe for use because they are natural [1, 4, 8, 9]. The chemical composition and phytochemical and macro/microelemental components of UNCP have been well studied using various methods [8–13]. UNCP contains about 1.9% theobromine, 0.21% caffeine, and micro- and macroelements where the level of the element copper, Cu^2+^, was 0.2984 ± 1.71 mg per 4 g, which exceeds the WHO permissible limits of 900 *μ*g [1, 9, 14, 15]. Luo et al. [16–18] advocate that high concentrations of the element copper tend to increase oxidative damage to lipids, proteins, and DNA. Cocoa beans, which are nonalkalized UNCP, have high percentages of polyphenols, epicatechins, and proanthocyanidins as compared to an alkalized cocoa powder [19].

Single (2000 mg/kg) and repeated oral treatment with UNCP (300 mg/kg, 900 mg/kg, and 1500 mg/kg, for two weeks) did not induce a visible toxicological effect on rat testis [15]. However, the existing research on the reproductive toxicity of UNCP is very scanty.

There is little evidence on studies on the reproductive toxicity of *Theobroma cacao* (UNCP). Potential teratogenicity and carcinogenicity have been reported in some studies while a decrease in body weight gain and epididymal weights have been observed in theobromine and high-dose cocoa-extract-treated animal groups [20–28]. Also, theobromine administered in feed severely impaired the reproductive capacity of female Swiss CD-1 mice, as evidenced by an increase in the number of lifeless pups per litter [29].

Herbal medicines and nutraceuticals are often used to manage various forms of reproductive health problems, especially in pregnancy. There has been increased use of such medications in women's health management [30–32]. Reports can be found on seed extracts of some medicinal plants in pregnant animals and also with a spectrum of adverse events [33–36]. Moreover, there have been other reports on reproductive toxicity associated with the use of some plant medicines used as food and nutraceutical while others also have been proven to have serious congenital malformations in pregnant mice [24]. UNCP in this instance is consumed regularly in various quantities among susceptible groups in the African population.

It is expected that UNCP may present no maternal and generative toxicity and reproductive performance in animals. This study, therefore, seeks to establish a preliminary genotoxic potential, establish the “no observed effect level” (NOEL)/no observed adverse effect level (NOAEL) of UNCP and possible reproductive toxic effect of UNCP on reproductive functions in both male and female rats during the stages of progenesis at very high doses.

## 2. Materials and Methods

### 2.1. Test Drug Preparation and Dosing

Brown Gold^®^ Natural Cocoa Powder obtained from Hords Company Ltd., Accra, Ghana (batch number BT620IT) and registered with the Ghana Food and Drugs Authority (FDA/DK06-070) was suspended in 1% starch. Fresh solutions of UNCP were always prepared prior to UNCP administration. Control animals received 1% starch suspension which is the solvent. UNCP was administered via intragastric route at a therapeutic dose (TD) of 900 mg/kg and a high dose of 9000 mg/kg (10 times higher than the therapeutic dose) in accordance with the guidelines for experimental preclinical research of new pharmacological substances [37].

### 2.2. Experimental Animals

Experiments were performed on healthy and matured (2.5–3.0 months) white Wistar rats (60 males and 120 females; weight 250–300 g) and male C57Bl/6 mice (30–45 g) obtained from the Experimental Biomodeling Department, Research Institute of Pharmacology and Regenerative Medicine (RIPRM) named after E. D. Goldberg, Tomsk, Russia.

#### 2.2.1. Animal Husbandry

All animals were housed in aluminium cages (VELAZ, 60 × 30 × 25 cm) with sterilised fine shavings under vivarium with ambient conditions of 20–240 C air temperature, relative humidity (50 ± 20%), air ventilation through HEPA filter, air exchange—12–15 volumes of room with ambient conditions of 12 h light/12 h dark cycle. Animals were fed on a granulated full-grain mixed feed from the veterinary and quality feed office, Tomsk, Russia. Noise and illumination levels were 50–55 dB and 300–350 lx, respectively. The range of concentrations of volatile substances (ammonia and carbon monoxide) as determined by the sensor was 0.4–1.0 mg/m^3^. Animals had free access to clean tap water (void of solid impurities, active chlorine, organic substances, and heavy metals) available in sterilised plastic bottles with metal nozzles-drinkers. Experiments were conducted in accordance with the rules adopted by the European Convention for the Protection of Vertebrates used for experimental and other scientific purposes. Also, institutional and specific national laws were strictly adhered to in conducting this research.

The experimental protocol was reviewed and approved by the ethical committee “Commission for control of content and use of laboratory animals” named after E. D. Goldberg, Russia, with protocol number P-55-G/12.10.17. These protocols were also conducted in accordance with the Organisation for Economic Cooperation and Development [38–40].

### 2.3. DNA Comet Assay

The alkaline single cell gel electrophoresis analysis (comet assay) has previously been used to study the potential of chemical-induced DNA damage in individual cells and genotoxicity in mice [40, 41].

In this study, male C57Bl/6 mice were randomly divided into four groups of 5 animals each and administered single doses of the test drugs orally as follows:

- The high-dose group: 9000 mg/kg UNCP
- The therapeutic dose group: 900 mg/kg UNCP
- The negative control group: solvent, 1% starch
- The positive control group: methyl methanesulfonate (40 mg/kg)

After treatment, male C57Bl/6 mice were euthanised by dislocation of the cervical vertebrae 3 hours after the single oral administration of test agents. The rectum, epithelium, liver, bone marrow, and kidney were isolated as quickly as possible to prevent invalid results from prolonged manipulations.

The epiphyses of the femurs were cut off and bone marrow cells washed from the diaphysis using 2 ml of phosphate-buffered saline (PBS) precooled to 4°C containing 20 mM EDTA-Na2 and 10% DMSO (pH 7.5). The liver, kidney, and rectum were homogenised in 3 ml of the same buffer. The tubes were held for 5 minutes at room temperature to precipitate large fragments, after which 1.5 ml of the top layer was transferred to a new tube. Cell suspensions in volumes of 60 *μ*l were introduced into a test tube with 240 *μ*l of 0.9% low-melting-point agarose solution (<42°C) in PBS heated to 42°C (microthermostat, “TERMIT”) and resuspended. Then, 60 *μ*l of the agarose solution with the cells was applied to precoated 1% versatile agarose slides that were covered with a coverslip and placed on ice. All subsequent operations were carried out in a dark room with yellow light. After hardening of the agarose (about 10 minutes), the coverslips were carefully removed, and micropreparations were placed in a glass cuvette (Schifferdecker type). This preparation was then poured into a 4°C lysis buffer (10 nM Tris-HCl (pH 10), 2.5 M NaCl, 100 mM EDTA-Na2, 1% Triton X-100, and 10% DMSO) and incubated for at least 1 hour. After lysis, the micropreparations were transferred to the electrophoresis chamber (Sub Cell GT, “Bio-Rad”). The chamber was filled with an electrophoresis buffer (300 mM NaOH, 1 mM EDTA-Na2, pH > 13). The prepared micropreparations were then incubated for 20 minutes to produce alkaline labile sites and alkaline DNA denaturation. Then, electrophoresis was performed for 20 minutes at a field strength of 1 V/cm and a current strength of ∼300 mA. The micropreparations were then transferred into a glass cuvette and fixed in a 70% solution of ethyl alcohol (for 15 min). After fixing, the micropreparations were dried and stained with SYBR Green I fluorescent dye (Sigma-Aldrich, USA) (1 : 10,000 in TE buffer with 50% glycerol) in the dark for 20 minutes and microscopy was performed. The analysis was carried out on a fluorescence microscope (Micromed 3 Lum, Russia) combined with a high-resolution digital camera (x200). The comet images were analysed using CASP software 8.0 (CASP, Wroclaw, Poland). The choice of this software was due to its free download availability on the Internet. As a measure of DNA damage, the percentage of tail DNA (tail DNA%) was used [38]. One hundred cells were analysed from each micropreparation. As a negative control, 2% starch (solvent) was used. Methyl methanesulfonate (methyl mesylate) 40 mg/kg, an alkylating agent and a carcinogen, which is also a reproductive toxicant served as a positive control. The criterion for toxicity was a statistically significant increase in the number of DNA comets, i.e., means of the treated groups and control groups [38]. Data were processed statistically with the help of the Wilcoxon–Mann–Whitney test. *P* values < 0.05 were considered significant.

### 2.4. Experimental Design of Reproductive Toxicology

Experimental animals in their progenesis stages were grouped in 2 batches of 90 animals each; the first batch “Experiment 1” (60 females and 30 males) was used to study female reproductive toxicology and the second batch “Experiment 2” (60 females and 30 males) was used to study male reproductive toxicology effect in females (see Table 1). All treated and untreated rats were subgrouped together for a 10-day cohabitation in a ratio of 2 females: 1 male [40, 42, 43] as follows.

### 2.5. Effect of UNCP on Reproductive Functions

Confirmation of the first day of pregnancy in the rats postcoitus was established with the help of a cytological evaluation of a vaginal smear and by observing a physical appearance of a vaginal plug [44, 45]. Seven pregnant females in each group were left for delivery, followed by a physical development of the offspring (survival index and body mass dynamics) until the 28th day of life. The rates were fixed at 4, 7, 14, 21, and 28th postnatal days of life (PND).

Euthanasia by carbon dioxide (CO_2_) overdose inhalation [46] was humanely performed on the rest of the females on the 17th to 20th day of pregnancy. An abdominal incision was made, and surgical opening was performed to expose Y-shaped uterus which was opened for pregnancy confirmation and easy determination of corpus luteum on the ovaries and the number of implantation sites and live and dead fetuses in the uterus. Based on the data obtained, the fertility index was calculated, as well as pre- and postimplantation mortality rates. In all, 120 female rats and 60 male rats were used (see Table 1).

#### 2.5.1. Historical Control Group

Embryonic death may occur before and after implantation in mammals, and some develop anomalies spontaneously. In this study, another control group of animals was created—the so-called “historical” control for purposes of analysing the indicators of embryonic death. Data from this “historical control group” were obtained in previous studies under similar conditions. The importance of using this group is well explained in the manual on preclinical studies in [42, 47].

### 2.6. Subacute and Reproductive Toxicity Assessment

To minimise the number of animals, we examined the 14-day potential toxicity of UNCP in male and female rats during the experiments on reproductive toxicity. Thus, common parameters (or main criteria) likely to give indications of the potential toxicity of UNCP in both male and female rats were assessed. Animals were observed daily throughout the study period, and clinical signs of toxidromes and mortality were observed. Changes in behaviour (agitation, lethargy, and hyperactivity), neurological changes (convulsions, tremors, muscle rigidity, and hyperreflexia), and autonomic signs (lacrimation, piloerection, pupil size, and unusual respiratory patterns) were also critically observed [48, 49].

Besides, decreases in the fecundity/pregnancy index, increased implantation losses (an increase in the rates of embryonic death in females), and a decrease in pub survival index and weight gain in the offspring were considered main criteria for reproductive toxicity.

### 2.7. Statistics

Data were exposed to variational statistics methods using IBM software and presented as mean ± SEM. The probability value (*P*) was calculated. The difference between the two compared values was considered significant if the probability of their identity was less than 5% (*P* < 0.05). In case of a normal distribution of traits for the statistical evaluation, the parametric Student's *t*-test was used. For large deviations of the characteristic distributions from the normal form for independent samples, a nonparametric criterion was used for the Mann–Whitney *U* test. To determine the reliability of the differences in qualitative indicators, the criterion of Fisher's angular transformation was used.

## 3. Results and Discussion

### 3.1. DNA Comet Assay

From Table 2, it is evident that UNCP at both therapeutic and high doses did not cause any damage to the DNA as compared to the positive controls.

**Figure 1:**
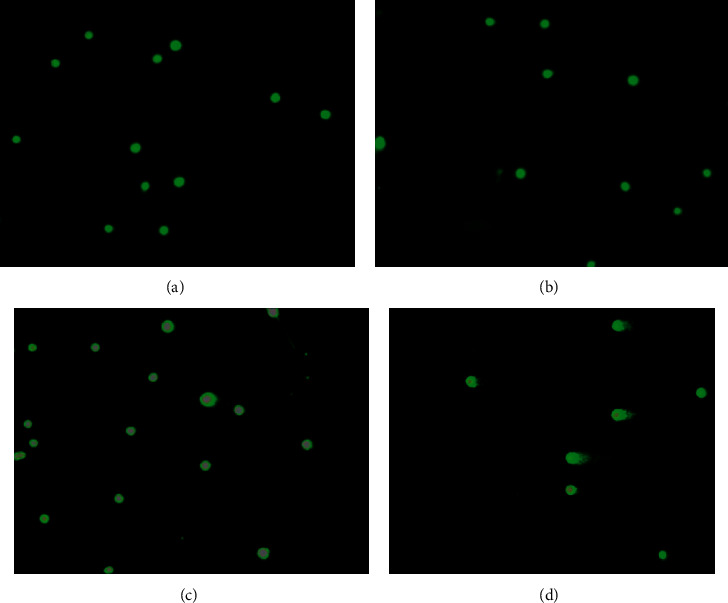
Photographs of cells analysed by comet assay analysis. The amount of migrated DNA is a measure of the extent of DNA damage. Bone marrow preparation x200 (% of DNA in the tail) of (a) UNCP 900 mg/kg, (b) UNCP 9000 mg/kg, (c) solvent, and (d) methyl methanesulfonate (MMS).

**Figure 2:**
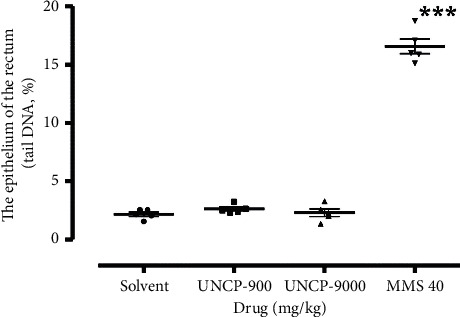
Changes in the levels of DNA damage (tail DNA, %) in the rectal epithelium of male C57Bl/6 mice. Note: ^*∗*^ means differences are significant when compared to the negative control (Wilcoxon–Mann–Whitney test).

**Figure 3:**
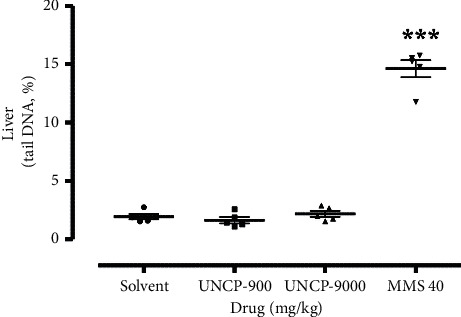
Changes in the levels of DNA damage (tail DNA, %) in the liver of male C57Bl/6 mice. Note: ^*∗*^ means differences are significant when compared to the negative control (Wilcoxon–Mann–Whitney test).

**Figure 4:**
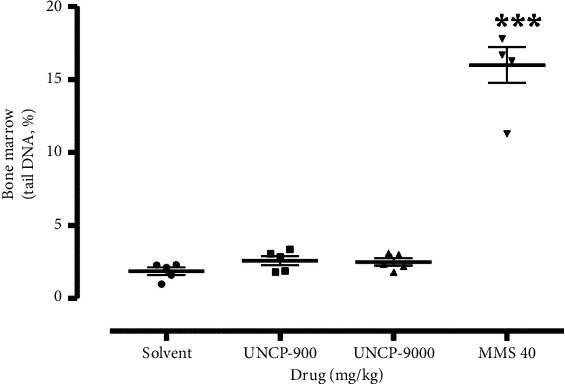
Changes in the levels of DNA damage (tail DNA, %) in the bone marrow of male C57Bl/6 mice. Note: ^*∗*^ means differences are significant when compared to the negative control (Wilcoxon–Mann–Whitney test).

The result is as presented in Figures 1–4.

From the scatter plots, it is evident that methyl methanesulfonate 40 mg/kg exerted the highest number of comet tails indicative of DNA damage in all the tissues analysed in comparison with the negative control (*P* < 0.05).

**Figure 5:**
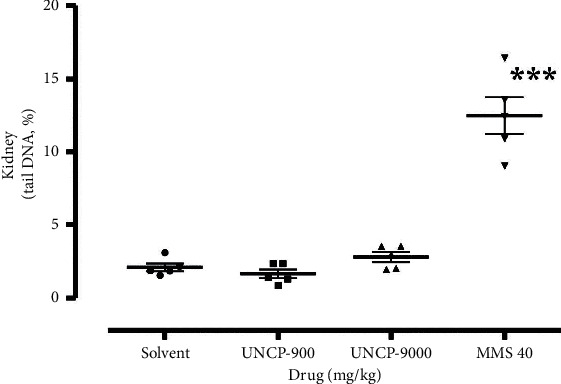
Changes in the levels of DNA damage (tail DNA, %) in the kidney of male C57Bl/6 mice. Note: ^*∗*^ means differences are significant when compared to the negative control (Wilcoxon–Mann–Whitney test).

As shown in Figures 2–5, UNCP (900 and 9000 mg/kg) did not induce a significant increase in the tail DNA% compared to the corresponding normal mouse tissues including the rectum, bone marrow, liver, and kidney.

Significant DNA destruction (tail DNA %) at 9000 mg/kg and 900 mg/kg UNCP occurred in the kidney (2.79 ± 0.35) and rectal epithelium (2.64 ± 0.17), respectively. Negative control tail DNA% values, in this case, corresponded to 2.17 ± 0.18 and 2.08 ± 0.27, respectively. It is interesting to note that the lowest tail DNA% value (lowest DNA destruction) was observed in the liver (1.64 ± 0.26) and kidney (1.63 ± 0.30) during 900 mg/kg UNCP administration. Negative control values corresponded to 1.95 ± 0.22 (21.7% increase) and 2.08 ± 0.27 (16% increase), respectively (Figures 2–5).

A very high tail DNA% (DNA breaks) was observed in all the organs during methyl methanesulfonate (methyl mesylate) administration (Figure 1(d)) compared with the negative control (1% starch) (Figure 1(c)). UNCP did not show any genotoxic potential (Figure 2) though, according to Guecheva et al. [17] and Chelomin et al. [18], high concentrations of the element copper have the tendency towards genotoxicity. However, high tail DNA% was observed in the rectal epithelium. In a toxicity study conducted, high GIT erosion was observed by Asiedu-Gyekye et al. [15]. UNCP has antioxidant and genoprotective activity (its ability to scavenge oxygen free radicals induced by mutagens). It is thus likely to prevent or reduce single-stranded DNA breaks.

### 3.2. Effects of UNCP on the Reproductive Function in Female Rats: “Experiment 1”

On the female reproductive function, it was established that the fertility index did not differ from that in the control. The number of corpora lutea in the ovaries, implantation sites, and live fetuses in the uterus and the indices of embryonic death in females receiving UNCP did not differ from the corresponding parameters of both vehicle and historical controls (Table 3).

#### 3.2.1. Effect of 15-Day UNCP Administration in Female Rats on Pregnancy Index

The results are presented in Table 3.

There was no significant change in gestational and pregnancy index from treated animals compared with the controls. The pregnancy index in the HD group corresponded to the historical controls while it was higher than the TD and vehicle control group.

#### 3.2.2. Effect of 15-Day UNCP Administration in Female Rats on Implantation

UNCP reduced preimplantation losses in both HD-treated group and TD-treated groups by 10.4% (*p* > 0.05) and 37% (*p* > 0.05), respectively, compared to the vehicle controls. Postimplantation losses were also reduced in both the HD- and TD-treated animals by 51.7% (*p* > 0.05) and 36.5% (*p* > 0.05), respectively, when compared with the vehicle control group.

Reduction in preimplantation loss was only observed with the TD-treated group (16%, *p* > 0.05) compared with the historical control group while postimplantation loss was observed only in the HD-treated group (14.4%, *p* > 0.05) compared to the historical control group. The progeny of animals of all groups were born without external pathological changes. Results are presented in Table 4.

Some medicinal plants have been found to modulate pregnancy in various ways by various mechanisms [51–53]. In our case, UNCP did not significantly influence pregnancy nor was it detrimental in both experiments 1 and 2. The fecundity or pregnancy index measures the female's ability to achieve pregnancy and is used in this study as a general indicator of reproductive toxicity because both sexes are treated [45, 54]. The gestation index measures the female's ability to maintain pregnancy, based on having delivered at least one live pub. In experiments 1 and 2, both the 15-day treated female groups and 48-day treated male groups did not result in any significant change in their pregnancy indices after cohabitation. The prognosis was however better with groups in experiment 1, the 15-day administered females.

Values are mean ± standard error of the mean, SEM (*n* = 9). Level of significance was established using statistical analysis of the data was done using the Mann–Whitney *U* test. Significant difference between dosed groups and control was evaluated by performing Student's one-tailed *t*-test. *p* values less than 0.05 were considered statistically significant.

#### 3.2.3. Effect of 15-Day UNCP Administration in Female Rats on Changes in Pub Weights

It was established that, over the entire period of observation, the dynamics of the rat weights in the progeny of females that received UNCP (before pregnancy) did not differ from that of the control animals (Table 5).

Between PND 4 and 28, the litter weights increased by 87.6% (*p* > 0.05) for females and 89.1% (*p* > 0.05) for males in the HD-treated animals compared to the vehicle control group which recorded 86.95% (*p* > 0.05) for females and 87.3% (*p* > 0.05) for males.

With the TD-treated group, there was an increase of 87.2% (*p* > 0.05) and 87.83% (*p* > 0.05) for both female and male groups, respectively, in the pub weights between PND 4 and 28.

#### 3.2.4. Effect of 15-Day UNCP Administration in Female Rats on Survival Index

Survival index of the offspring of treated females is presented in Table 6. Here, the administration of UNCP to female rats did not affect the survival of their offspring.

Between PND 1 and 4, the survival of the litters increased (HD by 3.3% (*p* > 0.05) and LD by 4.1% (*p* > 0.05)) compared to the vehicle controls. Between the PND 21 and PND 28, there was a 100% survival rate which signifies that UNCP did not have any adverse effect on offspring survival. Finally, on average, between days 1 and 28 of PND, UNCP increased survival rate by 10.82% (*p* > 0.05) among the TD-treated group.

No pub died PND 14–21 and PND 21–28 for both HD and TD groups, and pathological examination of pubs showed no abnormality.

Thus, the 15-day administration of UNCP to female rats did not lead to a change in reproductive function indicators and the offspring. The results, however, were not statistically significant (*p* > 0.05).

### 3.3. Effects of UNCP-Treated Male Rats on Reproductive Function Indicators: “Experiment 2”

The results revealed that the fertility index of the intact females mated with the treated males did not differ from that in the controls (Table 7). Moreover, there were no significant changes in their body weights as represented in Table 8.

The results revealed that the fertility index of the intact females mated with the treated males did not differ from that in the controls. The number of corpora lutea in the ovaries, implantation sites, and live fetuses in the uterus and the indices of embryonic death in females crossed with the 48-day UNCP administered males did not differ from the corresponding parameters of the animals in both the vehicle and historical control groups as evidenced in Table 9.

We wanted to observe how live pubs would be affected when male rats receive HD of UNCP for 48 days. Signs commonly associated with oral administration of high dose of any test drug were absent when 9,000 mg/kg UNCP was administered daily for 48 days which may indicate that UNCP is a relatively safe nutraceutical. This dose is ten times the recommended dose of 900 mg/kg (Asiedu-Gyekye et al., 2016). At this dose, it is evident that spermatogenesis was not affected, looking at the fertility index and implantation index (Tables 9 and 10). A possible explanation to the above effects of UNCP administration could be due to the high levels of polyphenols and proanthocyanidins, which have protective effects on the testes by activating Nrf2 signalling and prevent ovarian ageing in hens [56–58].

In a review paper, Roychoudhury et al. [59] speculate the adverse effects of high copper levels in interfering with both male and female developmental and reproductive functions and hampering embryo development in a dose-dependent manner. It is further believed that spermatozoa and testis are negatively affected by high concentrations of copper. We were expecting a negative influence of a high dose of 9000 mg/kg on the reproductive functions in both males and female rats, but our results revealed UNCP at this high dose did not have any noticeable adverse effect on the reproductive parameters.

In fact, the element copper deficiency has been found to be more detrimental when it is deficient than when in excess with regard to both reproductive and developmental effects [60]. Thus, one should have expected some morphological changes in the litters, but there were no visible morphological changes in the live pubs produced. Adeyina et al. [3] recommend a 200 g/kg hot cocoa bean shell to rabbits for an optimum physiological response. According to Asiedu-Gyekye et al. [15], 4 g of UNCP contains 0.2984 mg of the element copper. The human equivalent dose of 2000 mg/kg UNCP in rats corresponds to approximately 324.32 mg/kg human equivalent dose which is equivalent to 19,686.224 mg UNCP (approx. 8 teaspoonfuls if a teaspoonful of UNCP = 2.5 g) for an average human weight of 60.7 kg. Thus, the amount of copper contained in 19,686.224 mg UNCP will correspond to 1.469 mg (approximately 6 mg in the 9000 mg/kg dose), a value that is above the WHO guidelines (RDA of 900 *μ*g). Values are means; *p* > 0.05 was considered significant in all analysis.

#### 3.3.1. Effect of a 48-Day Administration of UNCP in Male Rats on Implantation

These results are presented in Table 9. There was reduced preimplantation loss of the TD group by 10.56% (*p* > 0.05) and HD group by 10.56% (*p* > 0.05) than the vehicle controls (21.04%, *p* > 0.05) and historical control (8.44%, *p* > 0.05). However, there was a surprise increase in postimplantation loss by 29.93% (*p* > 0.05) (HD), which occurred in the litters of 2 dams. At the same period, the TD recorded a postimplantation loss of 6.96% (*p* > 0.05) while the vehicle control and historical control recorded a loss of 2.87% and 8.49%, respectively.

The corpus luteum (yellowish/pinkish body on the ovaries) is formed from the thecal and granulosa cells of the postovulatory follicle. Being a transitory organ, a reduction in the number of corpora lutea or an increase in the pre- and/or postimplantation loss is considered an adverse reproductive effect. Disruption of early developmental processes may contribute to a reduction in fertilisation rate and increased early embryonic death prior to implantation [45]. This was calculated as the preimplantation losses. In our study, UNCP was found to have reduced preimplantation losses (Tables 4 and 9). Further, embryonic deaths after implantation and fetal deaths were expressed as postimplantation losses [46, 47].

A critical assessment of both experiments 1 and 2 on implantation revealed that UNCP administered to female rats for 15 days before mating significantly reduced pre- and postimplantation losses in both the HD- and TD-treated groups compared to both controls used. These implantation losses were observed in 1 out of 8 animals in the raw data.

A similar observation was made in male rats treated with UNCP for 48 days cohabiting with nontreated females. In this case, both pre- and postimplantation losses were reduced though, to a lesser extent than the treated female group. This may support UNCP's proimplantation potential [45–47]. The proimplantation effect of UNCP in treated females (Table 4) was supported by results on termed delivery of live pubs, implantation indices, pub weights, and survival indices (Tables 4, 6, 8, and 10). From PND 7 to 14, UNCP maintained the survival index by 100% (*p* > 0.05) with a zero death of pubs compared with the vehicle control group. Survival indices are primary endpoints which show the ability of offspring to survive postnatally to weaning. In this study, the survival index of treated animals did not differ from untreated ones. Further, in our study, low birth weight and impaired suckling ability were absent. Pup survival could also be influenced by litter size, lactational ability of the dam, maternal neglect, and acute intoxication during treatment [45] which were not affected by UNCP administration. In the latter case, continuous administration of UNCP at these higher doses was not accompanied by toxidromes in the administered animals. Reduction in preimplantation losses was observed in experiment 1. In fact, the relatively high postimplantation loss was observed in only 2 dams, a situation with an unclear explanation. Thus, UNCP could probably have a proimplantation potential.

According to Soffietti et al. [55], the inclusion of 1–1.5% theobromine in the diets of immature rabbits for 120 days caused severe and rapid mortality. Even among animals, some species such as dogs and horses appear to be far more sensitive to the adverse effects of the methylxanthine than others are. The reasons for this are not entirely clear but may lie in interspecies differences in the rate of theobromine catabolism.

It was also observed over the entire period that the litter weight changes in the progeny of females crossed with males that received UNCP did not differ from that of the control animals.

In this table, the number of live conceptuses or viable fetuses was reduced in the HD group (10.25 ± 2.24). The highest postimplantation loss of 29.93% (*p* > 0.05) was observed in the HD-treated group compared to the vehicle control group (2.87%, *p* > 0.05) and the historical group (8.49%, *p* > 0.05).

Preimplantation losses, however, were reduced in the HD-treated group (10.56%, *p* > 0.05) and TD group (9.55%, *p* > 0.05) compared to the vehicle control group (21.04%, *p* > 0.05). These losses were, however, higher than that observed in the historical control group, and these losses were observed in only 2 out of 8 animals. These observations may imply that the administration of UNCP to male rats at TD did not significantly affect spermatogenesis period. HD animals were slightly affected, and the rat progeny in all groups were born without external pathological changes.

#### 3.3.2. Effect of a 48-Day Administration of UNCP in Male Rats on Survival Index

Survival indices of offspring from females crossed with males that received 48-day UNCP are presented in Table 10. UNCP-treated male rats did not have a significant influence on the survival of their offspring. There was a marginal increase of 6.9% in the survival index at PND 1–28 at a dose of 9000 mg/kg and 8.61% at a dose of 900 mg/kg when compared with the vehicle control (*p* > 0.05). However, PND 7–14 was accompanied by a nonsignificant 0.5% decrease in survival index at both dose levels.

Thus, the 48-day administration of UNCP to male rats before crossing with intact females did not lead to significant changes in the indices characterising the state of the reproductive system and their offspring (*p* > 0.05).

PND of the pubs was not adversely affected by UNCP in both the 15-day treated female and 48-day treated male rats (Tables 6 and 10). The life of the offspring of both TD and HD by days 21 and 28 increased (*p* > 0.05) when compared to the vehicle controls. This further confirms an observation made by Tarka et al. [61]. Variations observed in these studies may be attributed to nonspecific maternal toxicity and may not be entirely attributed to UNCP though an unfavourable influence on spermatogenesis is not ruled out as reported by Tarka et al. [61]. The positive effects of UNCP contrary to expected adverse effects of individual constituents and elements might be due to the synergistic effects of the constituents and producing a very positive outcome on the reproductive processes.

### 3.4. Subacute Toxicity

Intragastric administration of UNCP to both male and female rats at doses of 900 mg/kg and 9000 mg/kg daily for 14 days produced no mortality during the observational period. Thus, the no-observable adverse effect could be estimated further to be around 9000 mg/kg. There were increased water intake and micturition in these animals.

The appearance and behaviour of the females who received the test substance for 15 days did not differ from that in the control (the rats were active and had a smooth wool coat and good appetite). There were no differences in mating behaviour exhibited by treated animals in the various groups and controls. Some male rats had frequent penile erections and sniffed the mouth and anogenital region of females while some of the treated females demonstrated enhanced wiggling of ears, hopping, and at times darting around the males. The aphrodisiac potential of *Theobroma cacao* has been reported which may explain the increased frequency of penile erections and frequency of mounting in the treated groups compared to the vehicle controls. The release of phenylethylamine, endorphins, serotonin, and zinc into the rat circulatory system may be responsible. The change in mating behaviour observed in most of the animals confirms other studies, reporting some aphrodisiac and mood-elevating effect of UNCP [1, 4, 50]. This was observed frequently in experiment 2.

This study has attempted to assess and resolve as a preliminary study the potential reproductive effects of UNCP and allay any fears of excessive consumption of UNCP by both males and females in their fertile years concerning potential reproductive toxicity. It also forms a basis for further research into the possible developmental and regenerative defects and changes in specific biomarkers during high consumptions of UNCP in Ghana and West Africa.

## 4. Definitions of Parameters of Reproductive Toxicity

Gestation index = % pregnancies yielding live litters.

Gestation index = (number of females with live born/number of females with evidence of pregnancy) × 100.

Survival index = (total number of live pubs (at designated time point)/number of pubs born) × 100.

Pregnancy index or fecundity: number of pregnancies being studied or pregnancy that has been included in the study; in effect pregnancy for sure.

Fecundity = number of pregnant females/number of females with confirmed mating × 100.

Fertility index was determined from the ratio of the number of pregnant females to the number of females cohabited/mated with males. That is percentage mating that resulted in pregnancy.

Implantation index was calculated by dividing the number of implantation sites by the number of corpora lutea.

Implantation index = number of implants/number of corpora lutea × 100.

Preimplantation mortality was determined by the difference between the number of yellow bodies in the ovaries and the number of implantation sites in the uterus.

Preimplantation loss = number of corpora lutea − number of implants/number of corpora lutea × 100.

Postimplantation mortality was determined by the difference between the number of implantation sites and the number of live fetuses.

Postimplantation loss = number of implants − number of viable fetuses/number of implants × 100.

Survival index = total number of live pubs (at designated time point)/number of pubs born × 100.

## 5. Conclusion

UNCP did not show any observable clinical toxidromes during subacute toxicity studies and is not likely to possess genotoxic potential from DNA comet assay. The administration of *Theobroma cacao* in the form of UNCP to female and male rats at doses of 900 mg/kg and 9000 mg/kg increased the survival index and did not adversely affect the fertility of animals, did not increase embryonic death, and does not have a toxic effect on their offspring. Thus, UNCP, a product from Accra, Ghana, is not likely to possess reproductive toxicity potential and has a broad reproductive safety profile.

## Figures and Tables

**Table 1:** Experimental design of reproductive toxicology.

Group	Number of animals		Notes
	Females	Males	
“Experiment 1”: reproductive function of females			
Cocoa HD	20	10	Female rats were orally administered UNCP at HD and LD for 15 days (duration of three oestrous cycles) before mating. Control animals received an equivalent volume of the vehicle (starch suspension) at the same time
Cocoa TD	20	10	
Control (solvent)	20	10	

“Experiment 2”: reproductive function of males			
Cocoa HD	20	10	Male rats were orally administered UNCP at HD and LD for 48 days (duration of one cycle of spermatogenesis) before mating. Control animals received an equivalent volume of the vehicle (starch suspension) at the same time
Cocoa TD	20	10	
Control (solvent)	20	10	
Total	120	60	[8, 9, 15, 43]

**Table 2:** Influence of UNCP on the level of DNA damage (tail DNA%) in tissues and organs of male C57Bl/6 mice.

Tissue				
Organ	The epithelium of the rectum (tail DNA, %)	Liver (tail DNA, %)	Bone marrow (tail DNA, %)	Kidney (tail DNA, %)
UNCP:9000 mg/kg (high dose)				
*M* ± SEM	2.32 ± 0.32	2.19 ± 0.26	2.49 ± 0.25	2.79 ± 0.35
UNCP:900 mg/kg (therapeutic dose)				
*M* ± SEM	2.64 ± 0.17	1.64 ± 0.26	2.58 ± 0.32	1.63 ± 0.30
The negative control (solvent, 1% starch)				
*M* ± SEM	2.17 ± 0.18	1.95 ± 0.22	1.86 ± 0.26	2.08 ± 0.27
Positive control (methyl methanesulfonate 40 mg/kg)				
*M* ± SEM	16.58 ± 0.63^*∗*^	14.63 ± 0.73^*∗*^	16.02 ± 1.23^*∗*^	12.49 ± 1.25^*∗*^

**Table 3:** Effect of UNCP on the fertility of female rats, when administered for 15 days before mating.

Group	Cohabited females (cohabited with males)	Number of pregnant females			Pregnancy index, %
		Number of dams delivered	Number of dams euthanised	Total	
Cocoa HD	20	7	9	16	80.00
Cocoa TD	20	7	7	14	70.00
Vehicle control	20	7	7	14	70.00
Historical controls	20	7	9	16	80.00

**Table 4:** Influence of UNCP on the reproductive function of female rats when administered for 15 days before mating.

Animal groupings	Number per female			Preimplantation loss, %	Postimplantation loss, %
	Corpora lutea	Implantation sites/number of implants	Viable fetuses		
Cocoa HD	13.22 ± 1.34	12.00 ± 1.54	11.22 ± 1.46	10.11 ± 5.60	7.27 ± 2.86
Cocoa TD	13.11 ± 0.72	12.33 ± 1.03	11.11 ± 1.27	7.09 ± 4.32	9.55 ± 6.74
Vehicle control	13.57 ± 0.84	12.14 ± 1.10	10.29 ± 1.19	11.28 ± 4.10	15.04 ± 5.47
Historical control	14.13 ± 0.88	13.00 ± 1.09	12.00 ± 1.21	8.44 ± 3.93	8.49 ± 2.80

**Table 5:** Changes in litter weights from female rats administered with UNCP for 15 days prior to mating.

Group	Sex	Postnatal days (PND)				
		4th day	7th day	14th day	21st day	28th day
Cocoa HD	♀	6.61 ± 0.23	9.58 ± 0.31	21.17 ± 0.63	30.04 ± 1.65	53.32 ± 3.14
	♂	6.67 ± 0.33	10.44 ± 0.31	22.64 ± 0.63	32.77 ± 1.77	60.95 ± 3.49

Cocoa TD	♀	7.44 ± 0.52	11.89 ± 1.01	23.14 ± 1.64	35.57 ± 1.89	58.41 ± 3.26
	♂	7.83 ± 0.35	11.79 ± 0.57	23.68 ± 1.25	36.40 ± 1.58	64.36 ± 4.09

Vehicle control	♀	8.27 ± 0.33	12.53 ± 0.66	25.13 ± 0.85	36.53 ± 1.19	63.42 ± 3.95
	♂	8.76 ± 0.44	13.12 ± 0.78	25.96 ± 1.15	37.06 ± 1.60	68.72 ± 2.32

**Table 6:** Survival index of pubs from female rats administered with UNCP for a 15-day period before mating (%).

Group	Postnatal days (PND)					
	1–4	4–7	7–14	14–21	21–28	1–28
Cocoa HD	92.32 ± 6.40	97.00 ± 2.03	94.39 ± 2.81	100.00 ± 0.00	100.00 ± 0.00	84.78 ± 6.79
Cocoa TD	93.06 ± 2.86	100.00 ± 0.00	100.00 ± 0.00	100.00 ± 0.00	100.00 ± 0.00	93.06 ± 2.86
Vehicle control	89.25 ± 7.21	100.00 ± 0.00	92.86 ± 5.17	98.41 ± 1.59	100.00 ± 0.00	82.99 ± 6.80

**Table 7:** Influence of a 48-day UNCP administration (in males) on the fertility of intact females during cohabitation (mating).

Group	Number of cohabited/mated females	Number of pregnant females			Index pregnancy, %
		Number of dams delivered	Number of dams euthanised	Total	
Cocoa HD	20	7	8	15	75.00
Cocoa TD	20	7	9	16	80.00
Vehicle control	20	7	10	17	85.00
Historical control	20	7	9	16	80.00

**Table 8:** Changes in pub weights from female rats crossed with males that received UNCP for 48 days prior to mating.

Litter size		PND				
Group	Sex	4th day	7th day	14th day	21st day	28th day
Cocoa HD	♀	8.63 ± 0.73	12.73 ± 0.92	24.83 ± 1.07	33.75 ± 2.55	60.35 ± 4.45
	♂	9.02 ± 0.75	13.29 ± 0.99	25.46 ± 1.13	35.10 ± 2.73	64.03 ± 5.27

Cocoa TD	♀	7.78 ± 0.81	10.84 ± 0.93	21.64 ± 0.99	30.68 ± 0.90	51.53 ± 1.95
	♂	8.42 ± 0.51	11.88 ± 0.77	22.95 ± 0.56	30.81 ± 0.87	50.34 ± 3.04

Vehicle control	♀	7.94 ± 0.68	12.38 ± 1.11	24.64 ± 2.68	34.29 ± 5.32	60.35 ± 9.10
	♂	8.60 ± 0.70	12.80 ± 1.10	25.93 ± 2.97	35.87 ± 5.37	62.95 ± 9.55

**Table 9:** Reproductive function of female rats crossed with males that received UNCP for 48 days before mating.

Doses	Quantity/amount per female			Preimplantation loss, %	Postimplantation loss, %
	Corpora lutea	Number of implants	Viable fetuses/full-term pups		
Cocoa HD	15.88 ± 0.52	14.13 ± 0.69	10.25 ± 2.24	10.56 ± 4.43	29.93 ± 13.98
Cocoa TD	14.89 ± 0.59	13.44 ± 0.69	12.44 ± 0.77	9.55 ± 3.65	6.96 ± 4.16
Vehicle control	15.90 ± 0.87	12.70 ± 1.36	12.30 ± 1.34	21.04 ± 7.47	2.87 ± 1.92
Historical control	14.13 ± 0.88	13.00 ± 1.09	12.00 ± 1.21	8.44 ± 3.93	8.49 ± 2.80

**Table 10:** Survival index of rats obtained from untreated female rats crossed with UNCP-treated males for 48 days before mating (%).

Group	PND					
	Days 1–4	Days 4–7	Days 7–14	Days 14–21	Days 21–28	Days 1–28
Cocoa HD	97.14 ± 2.86	96.43 ± 3.57	93.81 ± 4.74	100.00 ± 0.00	100.00 ± 0.00	90.00 ± 8.45
Cocoa TD	92.26 ± 3.58	97.51 ± 1.61	93.81 ± 4.74	98.70 ± 1.30	100.00 ± 0.00	84.11 ± 6.99
Vehicle control	99.05 ± 0.95	85.71 ± 14.29	98.81 ± 1.19	100.00 ± 0.00	100.00 ± 0.00	83.81 ± 14.09

## Data Availability

Raw data are available at School of Pharmacy, University of Ghana, and at the Goldberg Institute for Reproductive Toxicology. The raw materials are available at the School of Pharmacy, University of Ghana.

## Abbreviations

UNCP: Unsweetened natural cocoa powder
NOEL: No observed effect level
NOAEL: No observed adverse effect level
DNA: Deoxyribonucleic acid
HD: High dose
TD: Therapeutic dose
RIPRM: Research Institute of Pharmacology and Regenerative Medicine
PBMC: Peripheral blood mononuclear cells
OECD: Organisation of Economic and Co-operation and Development
DMSO: Dimethyl sulfoxide
EDTA: Ethylenediaminetetraacetic acid.
